# Observation of a superconducting glass state in granular superconducting diamond

**DOI:** 10.1038/s41598-019-40306-1

**Published:** 2019-03-14

**Authors:** G. M. Klemencic, J. M. Fellows, J. M. Werrell, S. Mandal, S. R. Giblin, R. A. Smith, O. A. Williams

**Affiliations:** 1 School of Physics and Astronomy, Cardiff University, Queen’s Buildings, The Parade, Cardiff, CF24 3AA UK; 2 School of Physics, HH Wills Physics Laboratory, University of Bristol, Tyndall Avenue, Bristol, BS8 1TL UK; 3 School of Physics and Astronomy, University of Birmingham, Edgbaston, Birmingham, B15 2TT UK

## Abstract

The magnetic field dependence of the superconductivity in nanocrystalline boron doped diamond thin films is reported. Evidence of a superconducting glass state is presented, as demonstrated by the observation of a quasi de Almeida-Thouless line in the phase diagram and a logarithmic time dependence of the magnetism. The position of the phase boundary in the *H*-*T* plane is determined from electrical transport data by detailed fitting to quasi-zero-dimensional fluctuation conductivity theory. This allows determination of the boundary between resistive and non-resistive behaviour to be made with greater precision than the standard *ad hoc* onset/midpoint/offset criterion. We attribute the glassy superconductivity to the morphological granularity of the diamond films.

## Introduction

The experimental observation of a proposed superconducting glass state was first made by Müller, Takashige and Bednorz in powder samples of La_2_CuO_4−*y*_:Ba^1^. The authors noted the existence of frustration in granular systems that gives rise to a “superconductive glass” that is equivalent to an XY spin-glass^2^, demonstrating the existence of degenerate states. In line with the essential features of the analogous spin-glass system, the authors reported a logarithmic decay of the remanent magnetization, and an irreversibility line–the boundary in the *H*-*T* plane between reversibility and irreversibility in magnetization–which obeyed ${H}_{irr}\propto {(1-T/{T}_{c})}^{3/2}$. This observed power law of the irreversibility line was likened to the de Almeida-Thouless line which separates the metastable and stable regions of the phase diagram in related models of spin-glasses^3^.

Similar behaviour regarding the irreversibility line and magnetic relaxation was subsequently observed in single crystal Y-Ba-Cu-O, and an alternative interpretation was proposed by Yeshurun and Malozemoff in the form of a thermally activated flux-creep model^4^. Tinkham extended this work and proposed a phenomenological model that included the effects of pinning and reproduced the observed power law^5^. Other theories purporting to explain the form of the irreversibility line in this kind of superconductor include vortex-lattice melting^6^ and the existence of a disordered vortex glass^7–9^ state arising from random pinning sites. Importantly, these competing theories share in common the idea of a randomly varying superconducting order parameter, leading to a ubiquitous 3/2 power law in the irreversibility line. We will therefore refer to this behaviour as “glassy superconductivity” without concern for the details of any specific theory.

Whilst the majority of reports of irreversible magnetic behaviour have focused on high-*T*_*c*_ compounds, Oppermann^10^ has pointed out that the signatures of a glassy state should also be expected in the vicinity of a metal-insulator transition, in both low- and high-*T*_*c*_ materials. Additionally, the existence of an irreversibility line has been studied in low-*T*_*c*_ type II materials such as Nb_3_Sn and Nb-Ti^11^, Nb^12^, MgB_2_^13^, PbMo_6_S_8_ and Ba_0.25_Pb_0.75_BiO_3_^14^. The irreversible behaviour in these metallic films has mainly been attributed to flux lattice melting.

In this paper, we describe the measurement of the magnetic field dependence of superconductivity in boron doped nanocrystalline diamond (BNCD) films, where the granularity may be controlled by the film thickness. We have found evidence of glassy superconductivity in the phase diagram, which we have confirmed with electrical resistivity and magnetic relaxation measurements. We observe a quasi de Almeida-Thouless-type line in *H*_*irr*_(*T*), and confirm the proposed glassy superconductivity by observing a logarithmic decay of magnetism over time. This glassy dynamics can be attributed to frustration in a system of weakly coupled superconducting clusters, each of which acts as an individual $U(1)\cong O(2)$ spin^2^. The BNCD films studied here have a granular morphology^15–18^ similar to sintered powders, and are close to a metal-insulator transition^19–21^, so that the observation of glassy superconductivity is perhaps not surprising. Nonetheless, this is a novel observation in BNCD films, made possible by detailed analysis of dimensional crossover in fluctuation conductivity^18^–*fluctuation spectroscopy*–which allows us to probe material properties that are governed by the morphological granularity of our films.

## Results

### Electrical resistance

**Figure 1:**
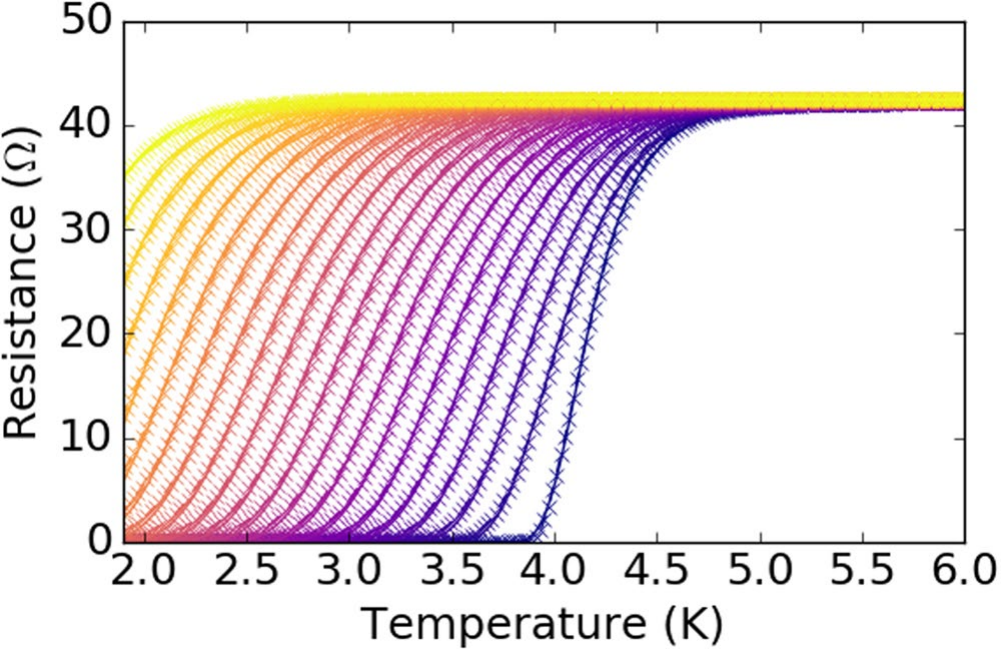
Resistance as a function of temperature for applied magnetic fields in the range 0–4 T in 0.2 T increments. Fit lines (solid) through the data follow the analysis technique discussed in the text.

**Figure 2:**
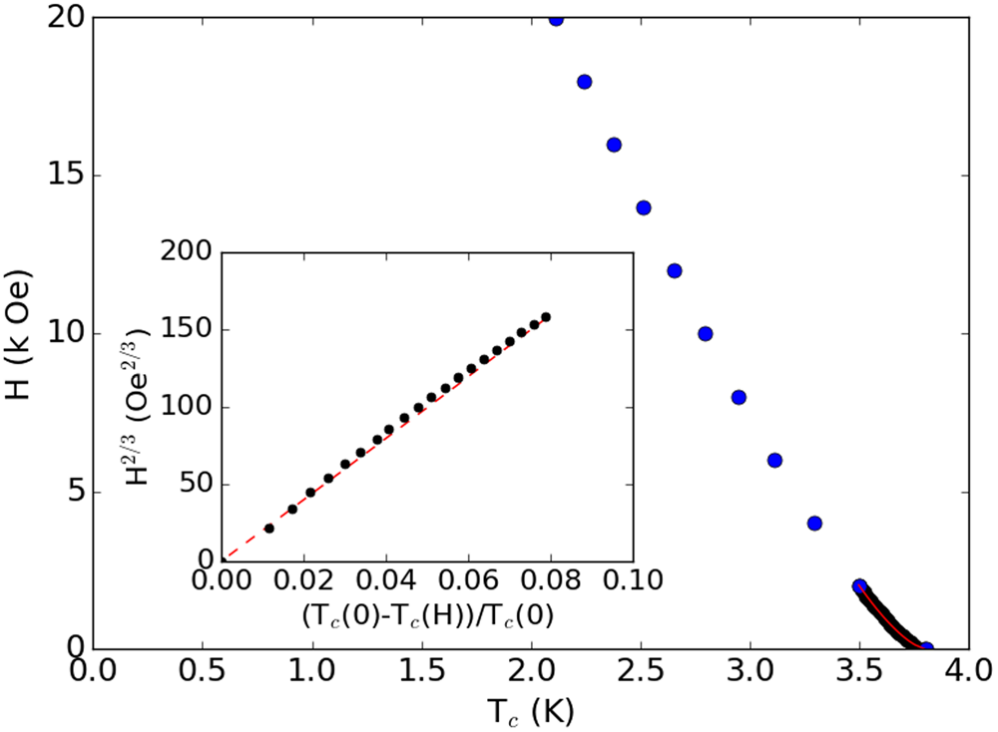
Magnetic field as a function of transition temperature for a 564 nm thick BNCD film. Inset: rescaled *H*(*T*) clearly showing the *H*^2/3^ power law behaviour.

Figure 1 displays the resistance as a function of temperature measured in an applied magnetic field in the range 0–4 T with field increments of 0.2 T. Closely spaced field increments of 0.02T (not shown in Fig. 1 for clarity, but present in Fig. 2) were also used below 0.2 T.

We are able to make a very precise determination of *T*_*c*_ in these films by fitting to the resistance data using our understanding of the fluctuation conductivity. In a previous work^18^, we have shown that the fluctuation contribution to the conductivity - that is the conductivity due to finite-lifetime Cooper pairs above *T*_*c*_ - exhibits a dimensional cross-over into a quasi-zero-dimensional regime. In the approach to the transition, this diverges as *σ*_fl_ ~ (*T* − *T*_*c*_)^−3^, in confirmation of a prediction by Lerner *et al*.^22^. In this region, the leading contribution to *σ*_*fl*_ comes from the lowest eigenvalue of the diffusion operator, −*D*∇^2^, inside the Cooper pair propagator for a single grain. In the presence of a magnetic field, this operator becomes $-D{(\nabla +2ie{\bf{A}}/\hslash )}^{2}$, and its lowest eigenvalue increases as a function of magnetic field. This correction to the pair propagator has the sole effect of reducing the transition temperature as a function of magnetic field, *T*_*c*_(*H*), so that *σ*_fl_ ~ (*T* − *T*_*c*_(*H*))^−3^.

We first extract the fluctuation conductivity from the resistance data by subtracting the normal state conductivity, which has the form ${\sigma }_{{\rm{n}}}=a+b\sqrt{T}$. Then, to determine *T*_*c*_, we fit the fluctuation conductivity to the quasi-zero-dimensional power law model, *σ*_fl_ ~ (*T* − *T*_*c*_)^−3^. This form holds for the widest part of the superconducting transition and allows us to obtain *T*_*c*_ as the single fitting parameter. After finding *T*_*c*_, we then further analyse the temperature dependence of the fluctuation conductivity, and observe two crossovers into three-dimensional intragrain and intergrain regions, both characterised by a *σ*_fl_ ~ (*T* − *T*_*c*_)^−1/2^ behaviour. The experimental *R*(*T*) curves can be well fitted using only the quasi-zero-dimensional dependence, *σ*_fl_ ~ (*T* − *T*_*c*_)^−3^, since this is valid for the widest part of the resistive transition.

The fitting of *T*_*c*_(*H*) using fluctuation conductivity is significantly more accurate than determining critical behaviour through the more common onset-midpoint-offset (90%–50%–10%) criterion^13^. Indeed, each *R*(*T*) trace in Fig. 1 is displayed along with the associated fit line, which shows an excellent agreement between the theoretical prediction and the experimental data. This agreement between theory and experiment strongly suggests that the broadening of the transition really is due to superconducting fluctuations rather than some other broadening effect coming from a distribution of local critical temperatures.

The resulting field induced transition temperature is shown in Fig. 2. It has been shown that this can be interpeted as the irreversibility line, *H*_irr_(*T*)^11,14,23,24^; as the magnetization becomes reversible, a zero voltage state is no longer supported, and a finite resistance is measured. Initially, at the higher temperature end of Fig. 2, there is a clear upturn in the *H*_irr_(*T*) curve, which closely follows the quasi de Almeida-Thouless *H*^2/3^ power law, as shown more clearly in the inset.

An important observation from the theory of Lerner *et al*.^22^ on which our theory is based is that for granular systems there are two distinct coherence lengths which must be considered; the coherence length associated with a Cooper pair which straddles a grain boundary through tunneling, *ξ*_*t*_, and that for a Cooper pair contained within a single grain, *ξ*_*g*_. These are related to the single-crystal diffusion constant, *D*, and the bulk effective diffusion constant, *D*_eff_, through the standard formula (see Varlamov *et al*.^25^ for example) ${\xi }_{g}=\sqrt{\pi \hslash D/8{k}_{B}{T}_{c}}$ and ${\xi }_{t}=\sqrt{\pi \hslash {D}_{{\rm{eff}}}/8{k}_{B}{T}_{c}}$. In our previous paper we used the location of cross-overs in the *R*(*T*) trace to estimate *D* ≈ 12 cm^2^/s and *D*_eff_ ≈ 0.6 cm^2^/s, which are comparable to measurements by more standard means. Based on these numbers we determine *ξ*_*g*_ ≈ 30 nm and *ξ*_*t*_ ≈ 7 nm. If we use the effective coherence length *ξ*_*t*_ to estimate the upper critical field using the standard formula ${H}_{c2}={{\rm{\Phi }}}_{0}/2\pi {\xi }^{2}$ we find an order of magnitude estimate 6.7 T which is comparable with estimates for similar samples^19^.

### Magnetic relaxation

We believe that we are in a regime where we observe glassy superconductivity in this system. To further explore this, we have also measured the relaxation of the magnetization, and observed the logarithmic decay that is expected from the analogous frustrated model.

The procedure for measuring the magnetic relaxation described by Yeshurun, Malozemoff and Shaulov^26^ was followed. The sample was cooled to the measurement temperature in zero applied field. A magnetic field smaller than the irreversibility field, but larger than the minimum sample magnetization, was then applied, followed by a step decrease that ensured a reversal of the flux profile. The values of these fields were determined by measurement of a magnetic hysteresis loop at the appropriate temperature. The magnetic moment was then monitored as a function of time by vibrating sample magnetometry, where the amplitude of sample translation was minimised to avoid possible field inhomogeneity.

**Figure 3:**
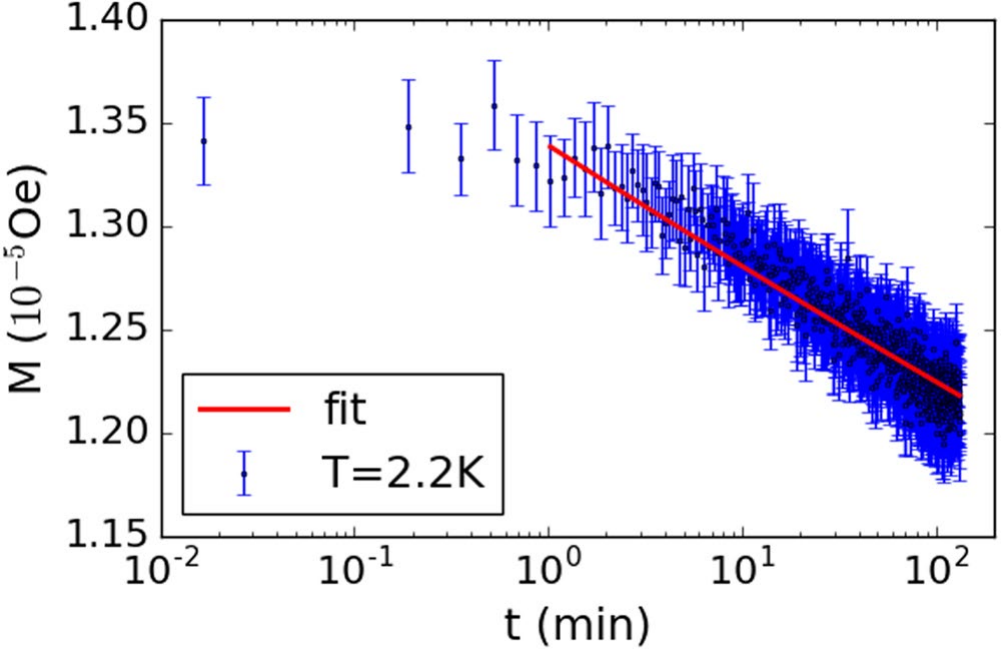
Magnetic relaxation measured at 2.2 K for a 564 nm thick BNCD film. The red line shows a linear fit of ln*M* versus ln*t*, performed after 1 minute to minimise the effect of instrumental transients. Similar logarithmic decay is observed over a range of temperatures up to *T*_*c*_.

The normalised magnetic relaxation at 2.2 K is shown in Fig. 3. For this measurement, after cooling in zero field, an initial applied field of 97 Oe was followed by a step decrease to 33 Oe. The red line is a linear fit to ln*M* as a function of ln*t*, which we later use to estimate the logarithmic derivative of the magnetic moment. We observe a logarithmic decay of the magnetic moment as expected for the analogous frustrated model to which we liken the observations.

**Figure 4:**
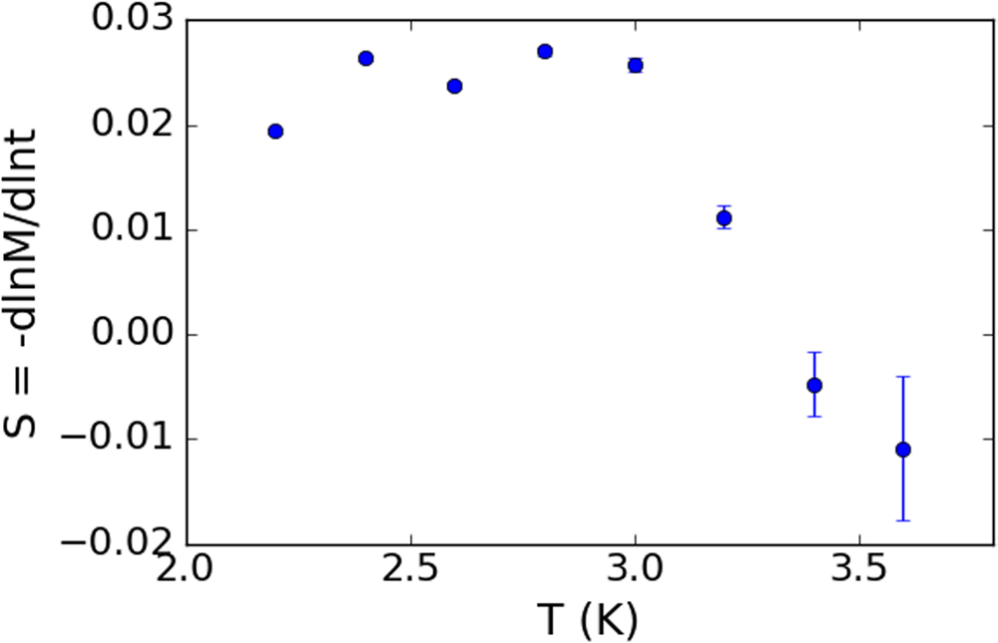
Normalized time-logarithmic slope of the magnetization, *S* = −*d*ln*M*(*t*)/*d*ln*t*, for a 564 nm thick BNCD film as a function of temperature.

The normalized time-logarithmic slope of the magnetization, $S=-\,d\,\mathrm{ln}\,M(t)/d\,\mathrm{ln}\,t$, is plotted as a function of temperature in Fig. 4. We observe the appearance of a plateau region for which S is stable at around 0.020–0.035. This is highly noteworthy because a universal plateau, with *S* in precisely this range of values, is observed in a number of experiments in Y-Ba-Cu-O and was explained by Malozemoff and Fisher^27^ as an effect of a vortex glass phase. An analogous plateau in *S* can be seen in other high temperature superconductors such as SmFeAsO_0.9_F_0.1_^28^ or ceramic (Bi, Pb)-2223^29^. At first sight it may seem odd that a universal feature associated with high temperature superconductors should be found also in diamond, a low temperature superconductor. However, the explanation put forward by Malozemoff and Fisher does not rely on the system in question being at high temperatures, but only on the existence of a dense environment of random pinning centres. The latter should certainly occur in any granular superconductor, including the BNCD films we consider here.

## Discussion

Generally, reports of the field dependence of the resistive transition in BNCD to date have shown that the transition width broadens with applied magnetic field; the onset of the superconductivity has been shown to be less sensitive to the field than the lower temperature tail in the resistance that grows with increasing field^30–32^. Scanning tunnelling microscopy of CVD-grown BNCD films well below *T*_*c*_ has shown that the temperature dependence of the energy gap in polycrystalline^33^ and epitaxial^34^ boron doped diamond samples both broadly agree with BCS-like superconductivity. In BNCD films, a strong correlation between the local superconductivity and the granular structure has been shown, with the plane of the grain boundaries approximately in the direction of film growth^33^. The energy gap showed a smooth variation across a grain, which is believed to be indicative of slowly varying doping concentration. The grain boundaries, however, are reported as having predominantly metallic-like conductance. It is therefore appropriate to consider our BNCD sample as a disordered collection of coupled superconducting grains. We thus interpret the results presented here as likely being due to a superconducting glass arising from frustration in a model of weakly linked superconducting clusters, as described by Ebner and Stroud^2^.

The samples studied here are not unique in terms of the existence of a quasi de Almeida-Thouless line in the *H*(*T*) diagram; there is some evidence in the literature that other BNCD samples show similar behaviour under the influence of a magnetic field^19,30,31,35^, but this feature has never before been investigated in detail. While discussion of, and experiments pertaining to, glassy states in superconductors are common for high-*T*_*c*_ compounds, they are also expected to be observable in granular materials, or materials near the metal-insulator transition^10^. The BNCD samples studied here fall into both of these categories, and so it is perhaps unsurprising that this glassy behaviour manifests itself here. Indeed, evidence for a vortex glass ground state has recently been reported in amorphous indium oxide films^36^, a similarly strongly disordered low *T*_*c*_ superconductor.

As an important side-note, it is common practice throughout the literature to calculate the coherence length in films such as these by comparing the *H*(*T*) curve to the Werthamer-Helfand-Hohenberg (WHH) theory^37^ of the upper critical field^31,38^. According to WHH theory, the *H*(*T*) curve should be linear near to *T*_*c*_, which is evidently not the case in samples displaying this glassy behaviour due to the curvature of the quasi de Almeida-Thouless line. It follows that fitting to WHH theory may not be a reliable way to determine the coherence length, and studies doing this may need to be reconsidered.

Finally, the signature of glassy superconductivity observed here has potential consequences for the use of BNCD in low temperature device engineering. The high Young’s modulus of diamond–even in the form of nanocrystalline films^39^–makes it a good candidate for the fabrication of high frequency nanoelectromechanical resonators with which to study the fundamentals of quantum mechanics in a macroscopic object^40,41^. Recently, an anomalous temperature dependence of the dissipation in a superconducting nanoresonator was observed, and was suggested to arise from the dynamical vortex behaviour below *T*_*c*_^42^. It is possible, therefore, that careful measurement of the temperature-dependent dissipation of a glassy superconducting nanoresonator^43^ would show complementary behaviour to that observed in this study.

## Conclusions

We have examined the magnetic field dependence of superconductivity in BNCD films. By determining the dependence of the transition temperature on increasing magnetic field, we have shown that the irreversibility field has the behaviour *H*_*irr*_(*T*) ∝ (1 − *T*/*T*_*c*_)^3/2^. This observed power law, coupled with a logarithmic decay of the remanent magnetization, is attributed to a superconducting glass state resulting from the morphological granularity of our BNCD samples.

## Methods

The BNCD film was grown on a 2″ diameter, 500 nm SiO_2_-buffered (100) silicon wafer by microwave plasma assisted chemical vapour deposition^44^. A dense population of nucleation sites on the substrate was obtained by ultrasonification in a monodisperse aqueous colloid of 5 nm diameter nanodiamond particles^45^, which has been shown to be essential in the production of uniform and fully coalesced nanodiamond films. During growth, the temperature of the substrate was maintained at ~720 °C by a plasma composed of 3% methane in hydrogen, with trimethylboron as the source of boron (B/C ratio of 12800 ppm). The chamber pressure was 40 Torr and the microwave power was 3.5 kW. The carrier concentration is estimated to be >1 × 10^21^ cm^−3^, with an upper limit of 2.3 × 10^21^ cm^−3^ assuming a 100% incorporation efficiency from the gas phase. The results presented in this paper focus on a BNCD film shown by scanning electron microscopy to have a thickness of 564 nm and a mean grain diameter of 102 ± 24 nm, though other sample thicknesses show a qualitatively similar behaviour. The estimated surface roughness is approximately 45 nm RMS. In previous work, we have shown that the shape and magnitude of the superconducting transition is unaffected by the surface roughness of the film as controlled through chemical mechanical polishing^46^.

All temperature dependent measurements relating to the magnetic field dependence of the superconductivity were made using a Quantum Design physical property measurement system. Resistance measurements were performed using silver paste contacts made to the sample surface in a four-wire Van der Pauw configuration. Vibrating sample magnetometry was used to measure the magnetization. All measurements were made with the magnetic field applied perpendicularly to the sample plane.
